# Animal Stress and Welfare During Transport and Slaughtering: An Outline for Future Policies

**DOI:** 10.3390/ani14213064

**Published:** 2024-10-24

**Authors:** Elbert Lambooij

**Affiliations:** Retired from Wageningen University & Research, Livestock Research, PO Box 9101, 6700 HB Wageningen, The Netherlands; bertlambooij@kpnmail.nl

## 1. Introduction

**Figure 1:**
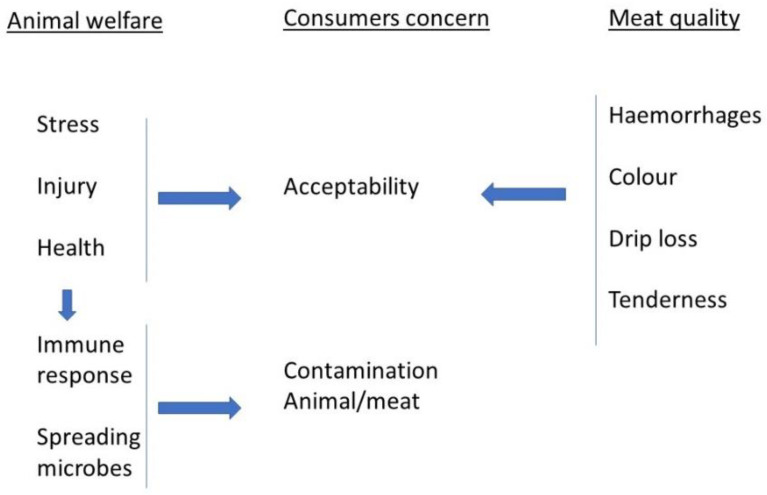
Welfare, concerns, and quality factors.

There is a lot of discussion about the transport of farm animals. The main issues include welfare, stress, legislation, consumer concern, and economic aspects. The main hazards identified for transported animals are common across all species. Hazards characterized as serious are inadequate ventilation, insufficient space allowance, transport duration, lack of appropriate food and water during transport, incorrect handling during (un)loading, poor fitness prior to transport, introduction of pathogens before and during transport, and the inappropriate application of resting periods during transport (Figure 1). Loading at the farm and unloading at the slaughterhouse have been considered one of the most stressful preslaughter events [1]. It is stated in the Treaty of Amsterdam [2] that all animals should be protected from avoidable sources of disturbance, pain, or suffering during transport, lairage, restraint, stunning, slaughter, or killing. The public pressure towards the welfare of animals during transport concerns the industry, and in response, they demand the introduction of new and more strict regulations. It is also stated that the management has to set up the welfare standards and monitor them via non-invasive tools and audits [3]. Here, the present progress in handling of farmed animals during transit and slaughterhouse is described.

## 2. Welfare and Stress

According to the Terrestrial Animal Health Code [4], animal welfare means the physical and mental state of an animal in relation to the conditions in which it lives and dies. The five components of animal welfare are freedom from hunger and thirst, discomfort, pain and injury or disease, fear and distress, and expression of normal behavior [5].

Until now, it is thought that a lot of behavior and cognition were exclusive to humans and some primates. Recently, it is considered that animals have knowledge of their own state, deal with their own knowledge, and evaluate the physiological state of their conspecifics [6]. Moreover, individual animals may have their own conscious profile since the possible presence of different profiles varies across species [3].

Death itself is not related to welfare, i.e., an animal’s welfare is not compromised by its dying. Events can influence welfare as long as the animal is alive, either at a farm or at an abattoir. Slaughter can be regarded as premature killing of healthy animals.

The adverse effects of animal welfare include several aspects, which can be assessed separately and possibly on different scales. Such components include the following: pain, fear, anxiety, frustration, behavioral disruption, malaise, discomfort [7]. These are all different aspects of welfare, which can be present at slaughter and killing, and which together, in some way, contribute to the total level of welfare [3]. Comparing values for the assessment and measurement of welfare is complicated as they are scored on different scales. The severity of these effects could be critical, severe, moderately limited, or negligible [8]. In the context of a relatively short slaughter process, some of the components seem more relevant to consider than others. The most relevant seem to be negative emotional states, like pain, distress, fear, and frustration, as well as physical injuries [9,10].

When an animal’s coping strategy cannot cooperate with changes in circumstances, we call this stress. In that case, the pituitary–adrenal system (glucocorticoids) and the sympathetic–adrenal medullary system (catecholamines) are activated, which results in behavioral and clinical deviations from normal functions; the control system overloads and fitness declines [11]. Tools to assess acute and chronic stress are used to observe behavior and measure heart rate, body temperature, and blood stress metabolites. Other measurements include skin damage score, meat pH, rigor mortis, temperature, color, water-binding capacity, and contamination [7].

## 3. Transport

Slaughter animals are transported at least once, but sometimes more, before they arrive at a slaughterhouse. A lot of animals are transported from the breeding farm to the fattening farm, and, when finished, to the slaughterhouse, sometimes via assembly places, which means one or more extra transports. The global animal slaughter was in 2022: 140 billion chickens, 1.5 billion pigs, 625 million sheep, and 300 million cows. Moreover, 90 million tons of live-weight fish are slaughtered [12].

According to an investigation by The Guardian, more than 20 million farm animals die during road transport from the farm to the slaughterhouse in the United States. Analysis of publicly available data shows that about 20 million chickens, 330,000 pigs, and 166,000 cattle were dead on arrival or died in lairage. The main causes are heat stress, especially in summer, freezing, or trauma [13]. In the Czech Republic, an investigation on the mortality rate after road transport showed pigs 0.065%, cattle 0.027%, and sheep 0.015%. The rate in laying hens was 0.507%, for broiler chickens it was 0.425%, for geese it was 0.003%, and for rabbits it was 0.199% [14].

An alternative for the transport of animals to a slaughterhouse is a mobile abattoir for partial or full slaughter. Because the slaughterhouse is transported to the animals, this is one method to avoid death in transport. Both stress and mortality are significantly lower. Problems related to this option include legislation and economic consequences [15,16]. In short, mobile slaughter would be too expensive for large scale use.

## 4. Lairage

At the arrival of the slaughterhouse, animals need to be checked for signs of poor welfare at farm level during loading and transportation. For instance, questions like space allowance or animals unfit to be transported are points to be assessed in the slaughterhouse that are related to the farm of origin; others, like some injuries in the animals, can be linked with the transportation conditions; and finally, during unloading, the condition of the ramp (slippering or not) and the animals (fearful, exhausted, and panting) must be assessed. When moved to lairage, the state of animals and handling of animals by the slaughterhouse personnel must also be considered. The lairage is the period between the entry of the animals into the resting pens (after being unloaded off the truck) until they are taken out of the pen to move to the stunning point. During lairage, animals are exposed simultaneously to a variety of stressors that may result in high levels of psychological and physical stress, thus compromising their welfare. These potential stressors can include fasting, mixing of unfamiliar individuals, handling by humans, exposure to a novel environment, noise, forced physical exercise, and in some cases extremes of temperature and humidity and water deprivation [17,18].

## 5. Stunning

It is necessary that slaughter animals need to be well restrained for an optimal stunning. Before restraining, animals are separated individually or in a group. In both situations, stress can occur. Individual restraining includes fixation by rope at the head of the animal or positioning in a gondola, V-type/rail conveyor, or in a (rotating) box. A group of animals can be driven automatically in a gondola. Control points for restraining are the size of the restrainer in relation to the species, throughput, duration, injuries, effect on bleedout, and worker safety [19].

In many countries, laws were introduced to avoid pain and suffering in animals during the slaughter process. Stunning is applied to induce unconsciousness and insensibility for the duration of death by exsanguination [20]. In general, unconsciousness means that the brain structures do not function. Stunning and killing methods for mammal, bird, and fish species include physical methods, electronarcosis, gas induction, and chemical methods. Physical methods include head percussion, brain penetration, spiking, decapitation, live chilling, and asphyxia. When electronarcosis is used, the electrodes can be placed on the head, or on the head and body, or include the entire body. The gasses used are carbon dioxide or nitrogen, and for chemical stunning, clove oil and Aqui-S™ are used [21].

Under laboratory conditions, the brain activity during stunning and thereafter can be measured with an EEG (electroencephalogram), VERs (visual evoked responses), VEPs (visual evoked potentials), or SERs (somatosensory evoked responses). Power spectral parameters can be used to quantify the depth of anesthesia. The EEG signals can be classified in delta (<4 Hz), theta (4 to 7 Hz), alpha (8 to 13 Hz), beta (13 to 32 Hz), and gamma (32 to 45 Hz) rhythms. During the delta and theta rhythms animals are considered to be unconscious [22]. In the slaughter line, important indicators for unconsciousness and insensibility are a physical collapse, rhythmic breathing, vocalization, eye reflex, wide open and relaxed eye and pupil, and blinking. Other indicators include the cognitive threat test, righting, nose pinch, and tongue hanging out [23,24].

Key factors that should be considered during bleeding are maintenance of devices, severed vessels, wound conditions, cardiac arrest, tonic and clonic muscle activity, and orientation of the carcass. When checks indicate that the animal shows signs of consciousness, intervention needs to be applied [23,24].

## 6. Recommendations for Developments

The state of consciousness of farmed animals continues to be a point of discussion. Further research regarding this state may result in improvements in legislation and management tools related to farms, transport devices, and slaughterhouses.

The environment of animals during transport can be measured using electrotechnical devices. However, the control, regulation, and adaptation during transport need improvement. Software and management tools can be developed to resolve welfare issues.

Indicators for unconsciousness and insensibility in the slaughter line are just clinical signs. A combination of electronic components may result in a device that measures the state of the brain, which refers to the subjective or inner qualitive experience of an animal. When the brain’s ability to integrate information is blocked or disrupted, the animal is unconscious [25].
